# Expression of concern: Synthesis of MA POSS–PMMA as an intraocular lens material with high light transmittance and good cytocompatibility

**DOI:** 10.1039/d3ra90083e

**Published:** 2023-09-05

**Authors:** Bailiang Wang, Quankui Lin, Chenghui Shen, Yuemei Han, Junmei Tang, Hao Chen

**Affiliations:** a School of Ophthalmology & Optometry, Eye Hospital, Wenzhou Medical University Wenzhou 325027 China lqk97531@126.com Chenhao823@mail.eye.ac.cn +86 577 88067962; b Wenzhou Institute of Biomaterials and Engineering Wenzhou 32500 China

## Abstract

Expression of concern for ‘Synthesis of MA POSS–PMMA as an intraocular lens material with high light transmittance and good cytocompatibility’ by Bailiang Wang *et al.*, *RSC Adv.*, 2014, **4**, 52959–52966, https://doi.org/10.1039/C4RA08060B.

*RSC Advances* is publishing this expression of concern in order to alert our readers that we are presently unable to confirm the reliability of the data presented in the article.

The Royal Society of Chemistry became aware of concerns about the reliability of the data presented in Fig. 8 and 12 of the paper.

The Royal Society of Chemistry has asked the affiliated institution, Wenzhou Medical University, to investigate this matter and confirm the integrity and reliability of the data in Fig. 8 and 12 of the paper. An expression of concern will continue to be associated with this manuscript until we receive information from the institution on this matter.

 

Laura Fisher

24th August 2023

Executive Editor, *RSC Advances*

## Supplementary Material

